# DACT1 Overexpression in type I ovarian cancer inhibits malignant expansion and cis-platinum resistance by modulating canonical Wnt signalling and autophagy

**DOI:** 10.1038/s41598-017-08249-7

**Published:** 2017-08-24

**Authors:** Ruo-nan Li, Bin Liu, Xue-mei Li, Liang-si Hou, Xiao-ling Mu, Hui Wang, Hua Linghu

**Affiliations:** 1 Department of Obstetrics and Gynaecology, the First Affiliated Hospital of Chongqing Medical University, Chongqing, 400016 China; 2 Experimental Research Centre, the First Affiliated Hospital of Chongqing Medical University, Chongqing, 400016 China; 3 Molecular Oncology and Epigenetics Laboratory, the First Affiliated Hospital of Chongqing Medical University, Chongqing, 400016 China; 4 Department of Pathology, the Basic Medical School of Chongqing Medical University, Chongqing, 400016 China; 5 Department of Gynaecologic Oncology, Anhui Provincial Cancer Hospital, Hefei, 230031 China

**Keywords:** Cancer therapeutic resistance, Ovarian cancer, Autophagy, Cell growth

## Abstract

Type I epithelial ovarian cancer (EOC) is primarily resistant to platinum-based chemotherapies and needs novel therapeutics. Given the aberrant Wnt activation in type I EOC and the involvement of Dapper1 Antagonist of Catenin-1 (DACT1) in Wnt signalling, the role of DACT1 in tumourigenesis of type I EOC was evaluated. Firstly, all tested EOC cell lines and primary EOC tissues, especially type I EOC, were observed to have significantly lower DACT1 expression than normal controls. Next, 3AO cells, which arise from a patient with primary mucinous EOC and express low endogenous levels of DACT1, were transfected with a lentivirus carrying full-length DACT1 (3AO-DACT1), grew slower and formed smaller tumours in nude mice compared to 3AO-NC. Furthermore, 3AO-DACT1 had lower levels of key mediators of canonical Wnt signalling, Dvl2 and β-catenin, GSK-3β with phosphorylated Ser9, and the Wnt/β-catenin target genes, with significantly lower nuclear β-catenin levels. Additionally, 3AO-DACT which contained higher levels of lipidated LC3 (LC3-II) and Beclin1, but lower levels of p62/SQSTM1, were more sensitive to cis-platinum. And chloroquine partially rescued its cis-platinum resistance. We identified DACT1 as a negative regulator in type I EOC, protecting against malignant expansion by inhibiting canonical Wnt signalling and cis-platinum resistance by regulating autophagy.

## Introduction

Epithelial ovarian cancer (EOC) is the fifth most common cancer worldwide and the most fatal gynaecological malignancy worldwide^1^. Approximately 21,290 women were diagnosed with ovarian cancer in 2015, and 14,180 died from the disease^2^. Despite fairly good initial responses to platinum-based chemotherapies, most patients suffer from relapse within 1 year of therapy^3, 4^, emphasizing the need for treatments that improve clinical outcomes.

EOCs are a heterogeneous group of tumours that can be classified into serous, mucinous, endometrioid, clear cell, and transitional and squamous tumours histologically^5^. Recently the dualistic model divided EOC into two categories, type I and type II^6^: type I EOCs include low-grade serous, low-grade endometrioid, clear cell, mucinous carcinomas, and transitional cell carcinomas while type II EOCs include the high-grade serous, high-grade endometrioid, and undifferentiated carcinomas. Type II EOC are most prevalent in postmenopausal women and account for 75% of ovarian cancers, causing 90% of deaths. In contrast, type I EOC are more common in younger patients, and characterized by voluminous and unilateral masses^7^. Type II EOC are usually initially relatively sensitive to platinum-based chemotherapy, but can develop resistance, while primary resistance to standard first-line platinum and Taxol chemotherapies is common in Type I EOC^8^, and mEOC was the very case in point. Thus the prognosis of advanced or recurrent mEOC is even worse than type II high grade serous cancer.

Wnt signalling has been reported to play key roles in the regulation of embryogenesis and tumourigenesis. The binding of Wnt ligands to membrane surface receptors Frizzled (Fz) and the co-receptors low density lipoprotein receptor-related proteins 5 and 6 (LRP5/6) are common upstream processes of canonical and noncanonical Wnt signalling pathways. Activation of these ligands recruits Dishevelled (Dvl) to Fz and Axin to LRP5/6 and causes disruption of the APC complex which is constituted by Axin, adenomatous polyposis coli, glycogen synthase kinase 3β (GSK-3β) and casein kinase 1, and consequently leads to accumulation of β-catenin. The accumulated β-catenin translocates into the nucleus and activates transcription of target genes such as C-myc^9^ and CyclinD1^10^ by interacting with the transcription factors, T-cell factor (TCF) and lymphoid enhancer binding factor (LEF)^11–13^. Aberrant activation of Wnt signalling has been implicated in the pathogenesis of EOC^14, 15^. However, the precise mechanism by which aberrant Wnt signalling contributes to EOC was not clear.

Dapper Antagonist of Catenin-1 (DACT1) was first identified as a protein interacting with Dishevelled-1 (Dvl), a central mediator in Wnt signalling. DACT1 controls Xenopus embryogenesis by antagonizing Dvl to modulate both canonical and noncanonical Wnt signalling^16, 17^. DACT1 induces lysosomal degradation of Dvl, and inhibits the transcription activity of Wnt-responsive reporters LEF and TCF^17^. However, the role of DACT1 in tumourigenesis was reported to vary between malignancies. Aberrant down regulation of DACT1 was observed in hepatocellular carcinoma, gastrointestinal stromal tumours and non-small-cell lung cancer (NSCLC)^18–20^, whereas in colon cancer and squamous cell carcinoma DACT1 was reported to be overexpressed^21, 22^. Recently, DACT1 has been reported to act as a positive regulator, promoting formation of the Beclin1-Vps34-Atg14L complex and enhancing autophagy^23^, and thus promoting Dvl2 ubiquitination^24^.

This study was designed to explore the role of DACT1 in EOC. We first found that DACT1 expression was depressed in EOC cell lines, and was especially lower in type I EOC, which include mEOC. This indicates DACT1 is dysregulated in mEOC. We found that overexpression of DACT1 in mEOC cell line 3AO reduced its expansion and cis-platinum resistance by regulating canonical Wnt signalling and autophagy.

## Results

### DACT1 expression was depressed in ovarian cancer cell lines and primary ovarian cancer tissues

**Figure 1:**
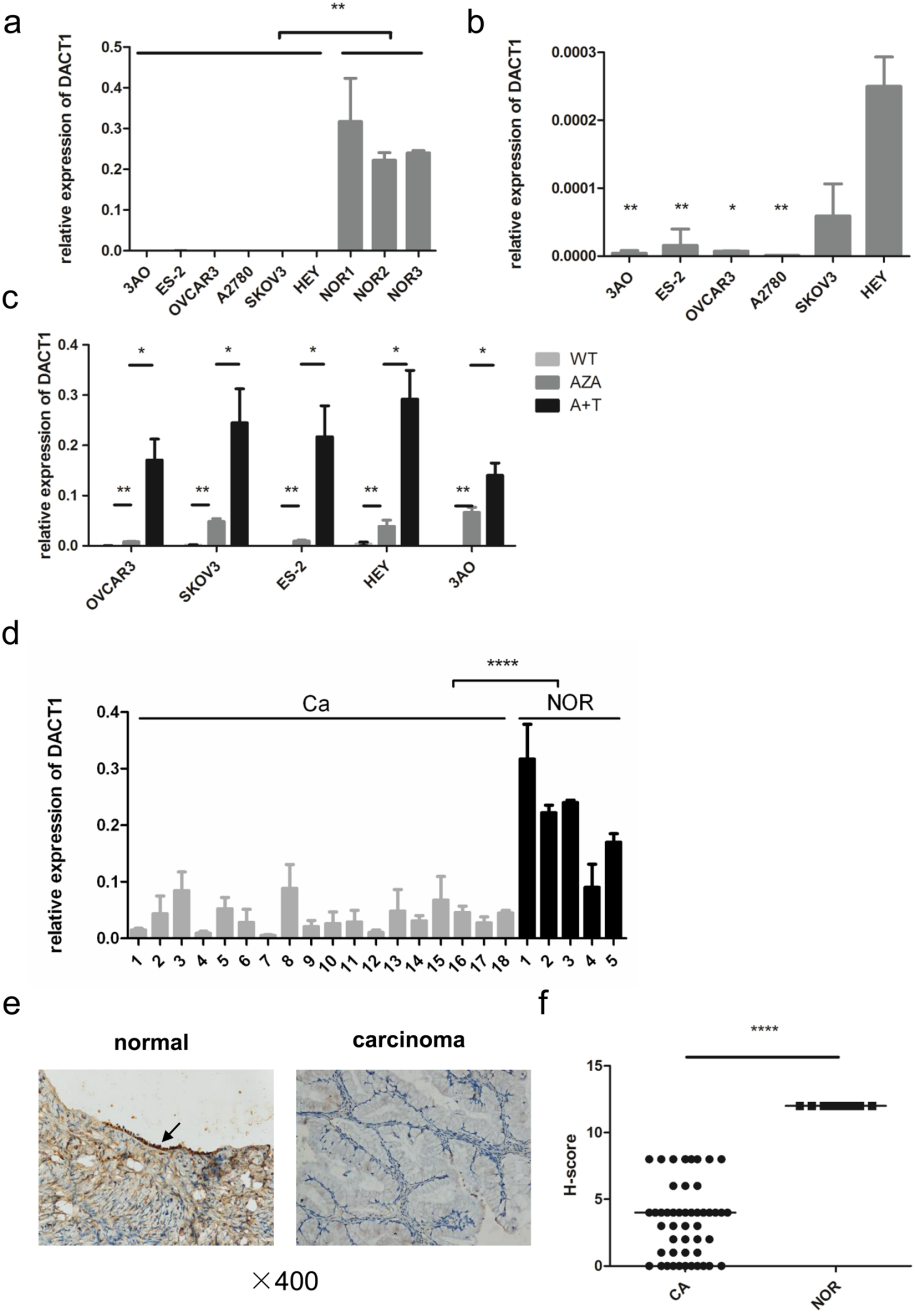
DACT1 expression in EOC cell lines and in type I EOC tissues. (**a**) DACT1 mRNA levels in EOC cell lines and normal ovarian tissues (NOR) were assessed by quantitative real-time RT-PCR. Expression was normalized to β-actin expression using the comparative CT-method (**P < 0.01). (**b**) DACT1 mRNA levels in EOC cell lines were assessed by quantitative real-time RT-PCR (*P < 0.05, **P < 0.01 compared with HEY). (**c**) Pharmacologic demethylation with Decitabine (Aza) in the presence or absence of trichostatin A (TSA) restored DACT1 expression in type I ovarian cancer cell lines. Ovarian cancer cells were treated with Decitabine for 72 h in the absence or presence of histone deacetylase inhibitor trichostatin A for 24 h, and DACT1 mRNA was detected by quantitative real-time RT-PCR3 (*P < 0.05, **P < 0.01). (WT: wild type; A + T: Aza with TSA). (**d**) DACT1 mRNA expression in type I primary EOC tissues and normal ovarian tissues was detected by quantitative real-time RT-PCR. Type I primary EOC tissue samples were obtained from 18 patients that underwent surgery. The normal ovarian tissues were obtained from patients with benign disease (adenomyosis, adenoma) who chose hysterectomy and oophorectomy (****P < 0.0001). (**e**) DACT1 protein expression in type I ovarian cancer and normal ovarian tissues was analysed by immunohistochemistry. Type I primary EOC tissue samples were obtained from 49 patients that underwent surgery. Benign and malignant ovarian mucinous tumour tissues were found in 5 patients, and a representative case was provided. Left: normal ovarian tissue, Right: mucinous carcinoma tissue, (which were captured in the same section). Images were digitally captured at a ×400 magnification ratio. (**f**) H-score was used to evaluate the level of DACT1 expression in type I ovarian cancer tissues and in normal ovarian tissues (****P < 0.0001). All the experiment was repeated at least three times.

To investigate whether DACT1 is implicated in ovarian cancer, we first examined its expression in several ovarian cancer cell lines by quantitative real-time RT-PCR. We found that DACT1 expression was significantly lower in all tested EOC cell lines (SKOV3, ES-2, OVCAR3, 3AO, A2780 and HEY) than in normal ovarian tissues (Fig. 1a). Interestingly, compared with that in type II EOC, the level of DACT1 expression was significantly lower in type I EOC, including 3AO, OVCAR3, ES-2 and A2780 (Fig. 1b), among which 3AO was reported arising from a patient with primary mEOC^25^.

A typical CpG island was found to span the proximal promoter and exon 1 regions of the DACT1 gene^26^. We thus speculated that DACT1 promoter methylation may be responsible for DACT1 expression depression in EOC cell lines. As indicated in Fig. 1c, after incubation with Decitabine in the presence or absence of histone deacetylase inhibitor TSA, the DACT1 expression in all EOC cell lines was dramatically restored, suggesting that methylation of the DACT1 promoter may contribute to DACT1 silencing in EOC (Fig. 1c). Similar results were observed by semi-quantitative RT-PCR (Supplementary Figs S1 and 2).

To investigate the clinical relevance of these findings, we measured DACT1 mRNA levels in type I ovarian cancer tissues and normal ovarian tissues. Type I primary EOC tissue samples were collected from 18 patients during surgery. As expected, DACT1 expression was significantly lower than in normal ovarian tissues (P < 0.0001) (Fig. 1d).

We further analysed the expression of DACT1 protein in 49 cases of type I EOC tissues and 10 cases of normal ovarian tissues using immunohistochemistry (IHC). The intensity of DACT1 staining in EOC tissues significantly is weaker than in normal control tissues (P < 0.0001). Interestingly, we found that benign, borderline and malignant ovarian mucinous tumour tissues were co-existed in five cases of mEOC specimens. Staining of DACT1 was more intense in benign lesions than in cancer tissues (Fig. 1e,f). Furthermore, in the sections of five cases with mEOC, the expression of DACT1 in benign lesion was observed significantly stronger than that in cancer tissues on the same section (Supplementary Fig. S3). Such stepwise change of DACT1 expression from normal to benign to malignant tissue further supports the role of DACT1 in the etiopathogenesis of type I EOC. Depressed expression of DACT1 in malignant cells further supports the etiopathogenetic role of DACT1 in mEOC.

### DACT1 inhibits ovarian cancer cell growth

**Figure 2:**
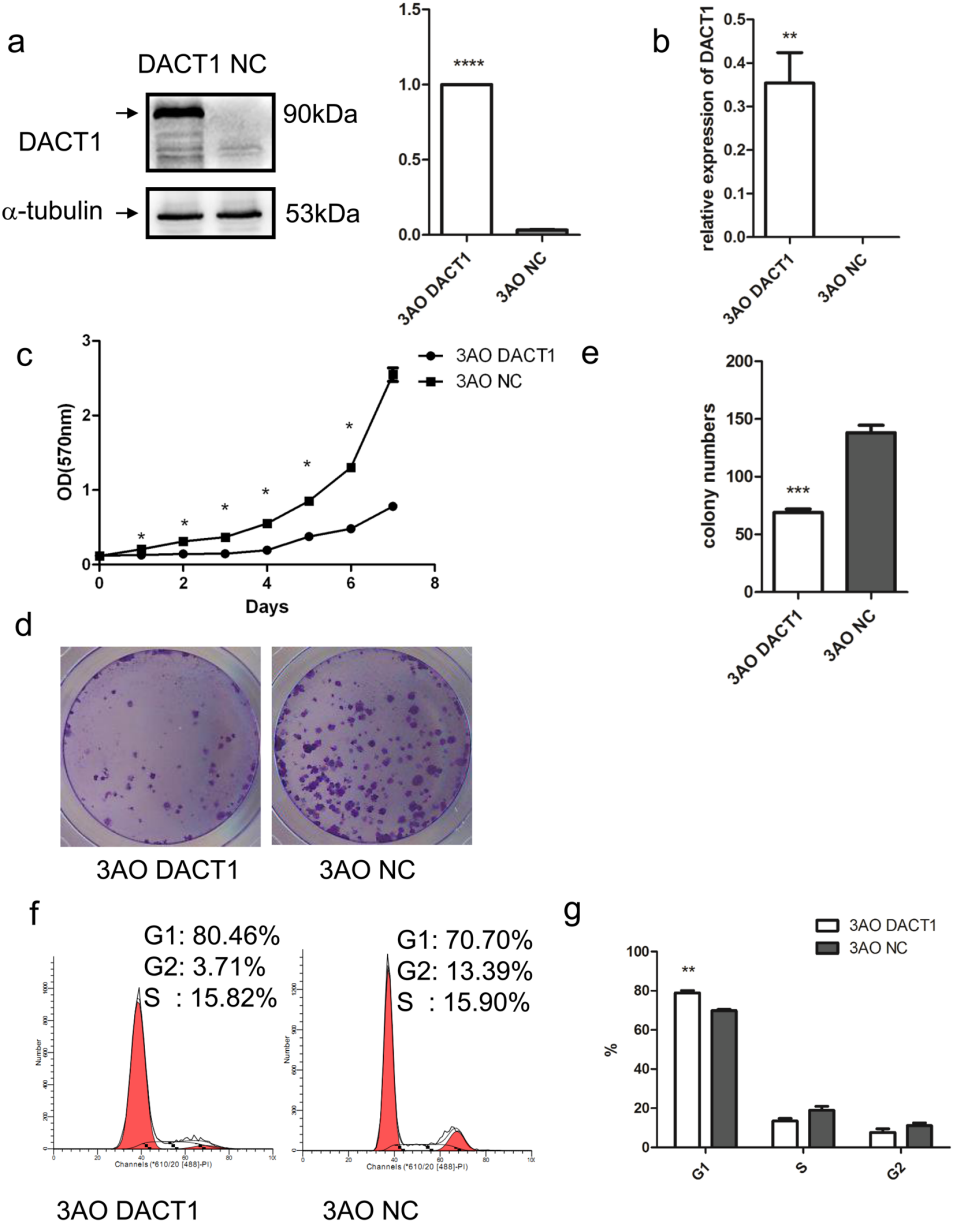
Overexpression of DACT1 inhibition of cell growth and clonogenicity in 3AO cell line. (**a**) Representative Western blots of negative control-transfected (NC) and DACT1-transfected 3AO cells (DACT1), a typical type I EOC cell line with clear genetic background which arises from a patient with primary mucinous adenocarcinoma. α-tubulin was used as the loading control (****P < 0.0001). (**b**) The level of DACT1 mRNA expression in 3AO-DACT1 cells and 3AO-NC cells was detected by quantitative real-time RT-PCR (**P < 0.01). (**c**) Growth curve of 3AO-DACT1 cells and 3AO-NC cells (p < 0.05, paired t test). Cell numbers were assessed by MTT assay from days 0 to 7. (**d**) and (**e**) The effect of DACT1 overexpression on 3AO cell colony formation. 3AO-DACT1 or 3AO-NC were incubated for 12 days and colonies of over 50 cells were counted after staining with crystal violet. (***P < 0.001). (**f**) The effect of DACT1 on 3AO cell cycle was assessed by flow cytometry (**P < 0.01). All the experiment was repeated at least three times.

To elucidate the function of DACT1 in mEOC, we examined the effect of DACT1 expression on the growth characteristics of mucinous ovarian cancer cells using colony formation and growth curve assays. 3AO was isolated from a patient with primary mucinous adenocarcinoma^25^ and chosen for its histologic type (Supplementary Fig. S4). 3AO cells, which express very low endogenous levels of DACT1, were transfected with a lentivirus carrying full-length DACT1 (3AO-DACT1) or a control vector (3AO-NC). Stable expression of DACT1 was confirmed using western blot and quantitative real-time RT-PCR (Fig. 2a,b). MTT assay was used to assess proliferation. The proliferation of 3AO-DACT1 was significantly slower than 3AO-NC (P < 0.001) (Fig. 2c). Consistent with this finding, 3AO-DACT1 formed significantly fewer and smaller colonies than 3AO-NC (P < 0.001) (Fig. 2d,e).

Next we assessed the effect of DACT1 on cell cycle in 3AO cells. Cells were synchronized at the G0/G1 phases by serum starvation for 16 h, then cultured in complete medium for 72 h and cell cycle was assessed by flow cytometry. 3AO-DACT1 progressed from G1 to S phase at a slower rate than 3AO-NC, and thus were arrested in the G0/G1 phase (Fig. 2f,g). Considering that apoptosis also affects tumour growth, we assessed the apoptosis rate of 3AO-DACT1 and 3AO-NC, however, there was no significant difference between the two groups (data not shown).

### DACT1 inhibits ovarian tumourigenesis *in vivo*

**Figure 3:**
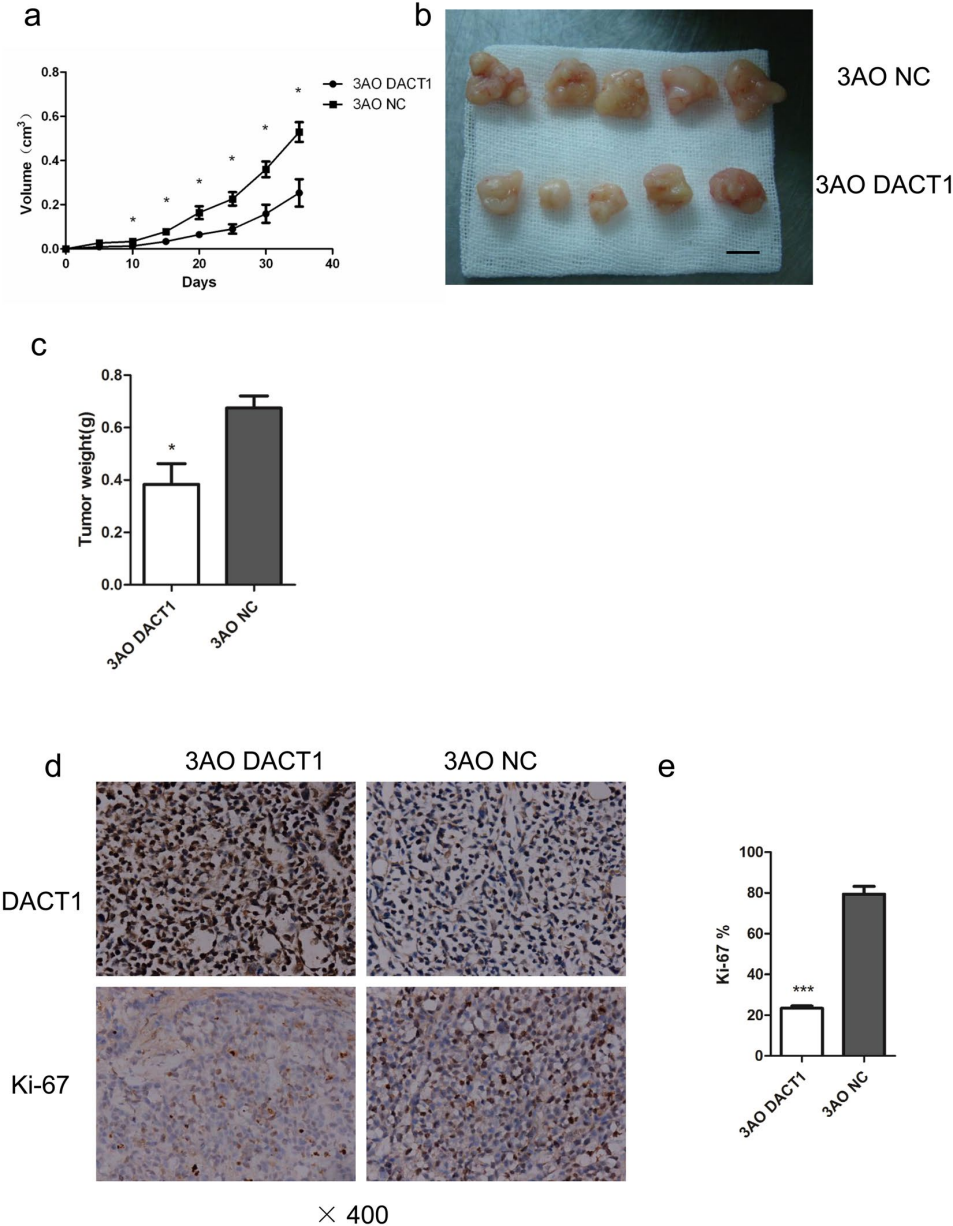
DACT1 inhibits ovarian tumourigenesis *in vivo*. (**a**) 5 * 10^6^ 3AO-DACT1 or 3AO-NC cells were injected into the subcutaneous tissue of each nude mouse (n = 5 animals per group). Average tumour volume was assessed weekly (*P < 0.05, paired t test). (**b**) Representative images of xenografts five weeks after injection. (**c**) Five weeks after injection, xenograft tumours were resected and measured. The average weight of the DACT1 over-expressing xenograft tumours was significantly lower than that of control xenograft tumours (*P < 0.05). (**d**) and (**e**) Proliferation indexes of DACT1 over-expressing and control xenograft tumours were evaluated by Ki-67. Cell proliferation was significantly higher in control xenograft tumours than DACT1 over-expressing tumours (***P < 0.001).

To further elucidate the tumour-suppressive function of DACT1 *in vivo*, 3AO-NC or 3AO-DACT1 cells were transplanted into five nude mice. Tumour size was measured and the growth-curves of tumours were observed (P < 0.05) (Fig. 3a), then tumours were excised for immunohistochemistry. The graft tumours from 3AO-NC injected-animals were significantly larger than those of 3AO-DACT1 (Fig. 3b). The mean tumour weight was significantly lower in the 3AO-DACT1 group (0.384 ± 0.176 g) than the 3AO-NC group (0.675 ± 0.102 g, P < 0.05) (Fig. 3c).

Proliferation of xenograft tumours was also assessed by Ki-67 assay. Consistent with the results obtained *in vitro*, DACT1-expressing tumours displayed significantly fewer proliferative cells than control tumours (Fig. 3d). The tumour-suppressive function of DACT1 was also confirmed by intraperitoneal injection of 3AO-DACT1 or 3AO-NC cells (Supplementary Fig. S5a,b). These findings further implicated that DACT1 may suppress the proliferation of 3AO.

### DACT1 regulates cell cycle by attenuating β-catenin-dependent Wnt signalling

**Figure 4:**
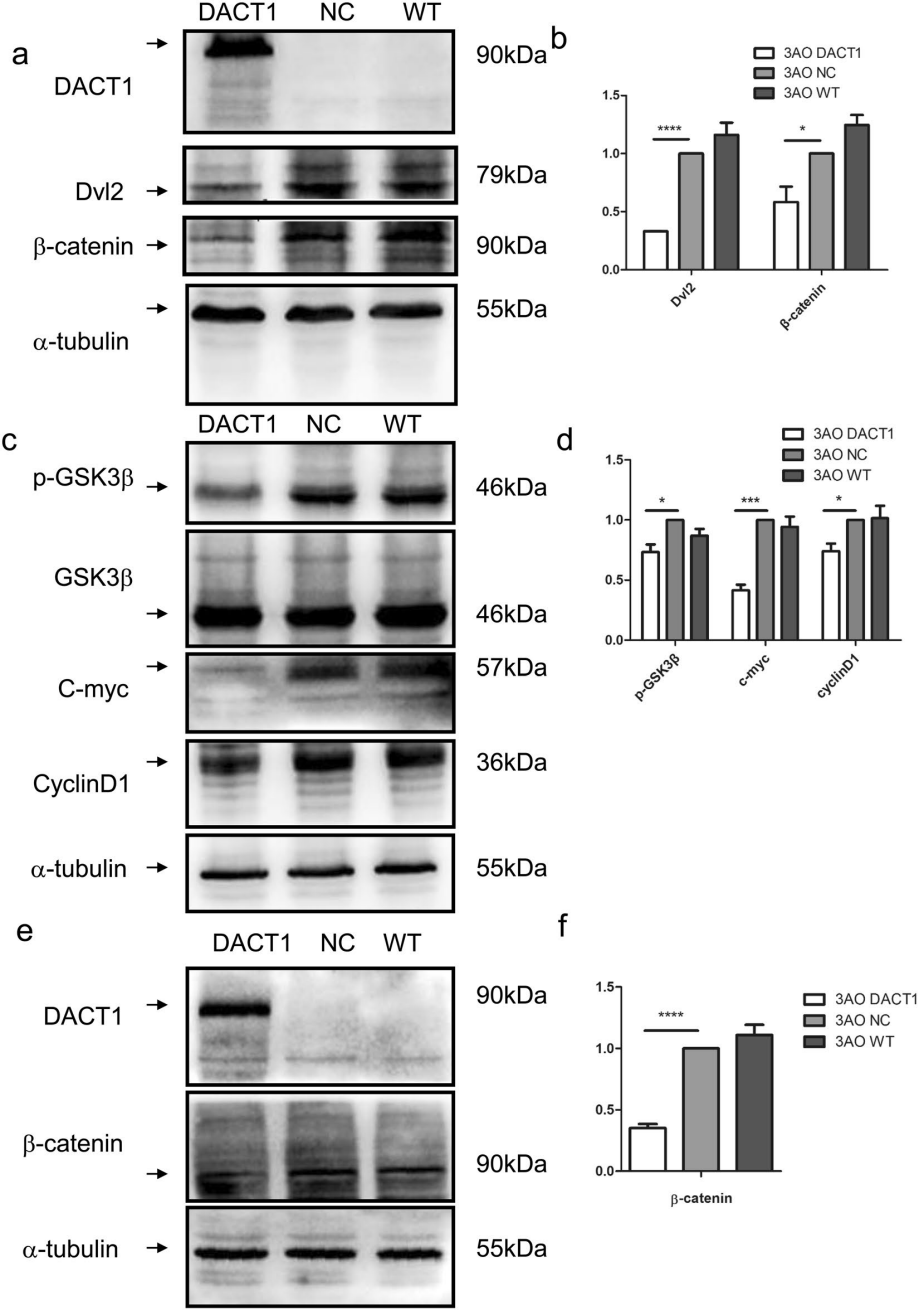
DACT1 suppresses Wnt/β-catenin signalling by inducing Dvl2 degradation and β-catenin nuclear translocation. (**a**) and (**b**) The Dvl2 and β-catenin content of 3AO cells was significantly lower in cells overexpression DACT1. Experiments were performed in triplicate and grey values were measured by Fusion (*P < 0.05, ****P < 0.0001). (NC: negative control; WT: wild type). (**c**) and (**d**) Overexpression of DACT1 also significantly reduced cellular levels of GSK-3β with phosphorylated Ser9, and the Wnt/β-catenin target genes, CyclinD1 and C-myc. Grey values collected by Fusion were analysed (*P < 0.05, ***P < 0.001). (**e**) and (**f**) Nuclear protein was extracted and demonstrated that β-catenin translocation to the nucleus was significantly decreased by overexpression of DACT1. α-tubulin was used as the loading control. Grey values collected by Fusion were analysed. (****P < 0.0001). All the experiment was repeated at least three times.

It is reported that canonical Wnt signalling can affect the EOC growth. To elucidate the mechanism by which DACT1 suppresses ovarian cancer cell growth, we assessed the levels of the key mediators of canonical Wnt signalling, such as Dvl2 and β-catenin, in 3AO-DACT1 (Fig. 4a,b). We found that cellular levels of Dvl2 and β-catenin were lower in 3AO-DACT1 than 3AO-NC, and cellular levels of GSK-3β with phosphorylated Ser9, and the Wnt/β-catenin target genes, CyclinD1 and C-myc, were lower in 3AO-DACT1 than 3AO-NC (Fig. 4c,d).

The subcellular location of β-catenin was assessed to investigate whether DACT1 influenced the nuclear translocation of β-catenin (Figs 4e,f and S6). Nuclear β-catenin levels were significantly lower in 3AO-DACT1 than 3AO-NC. These results further support the theory that DACT1 regulates the subcellular localization of β-catenin by stimulating GSK-3β.

### DACT1 overexpression induces autophagy and sensitizes 3AO to cis-platinum

DACT1 was found to act as a positive regulator to promote the formation of the Beclin1-Vps34-Atg14L complex and enhance autophagy^23^, and autophagy has been reported to be a putative mechanism of resistance to cis-platinum (CDDP) recently^27, 28^. These findings prompted us to explore whether DACT1 could influence the cis-platinum sensitivity of type I EOC by activating autophagy.

**Figure 5:**
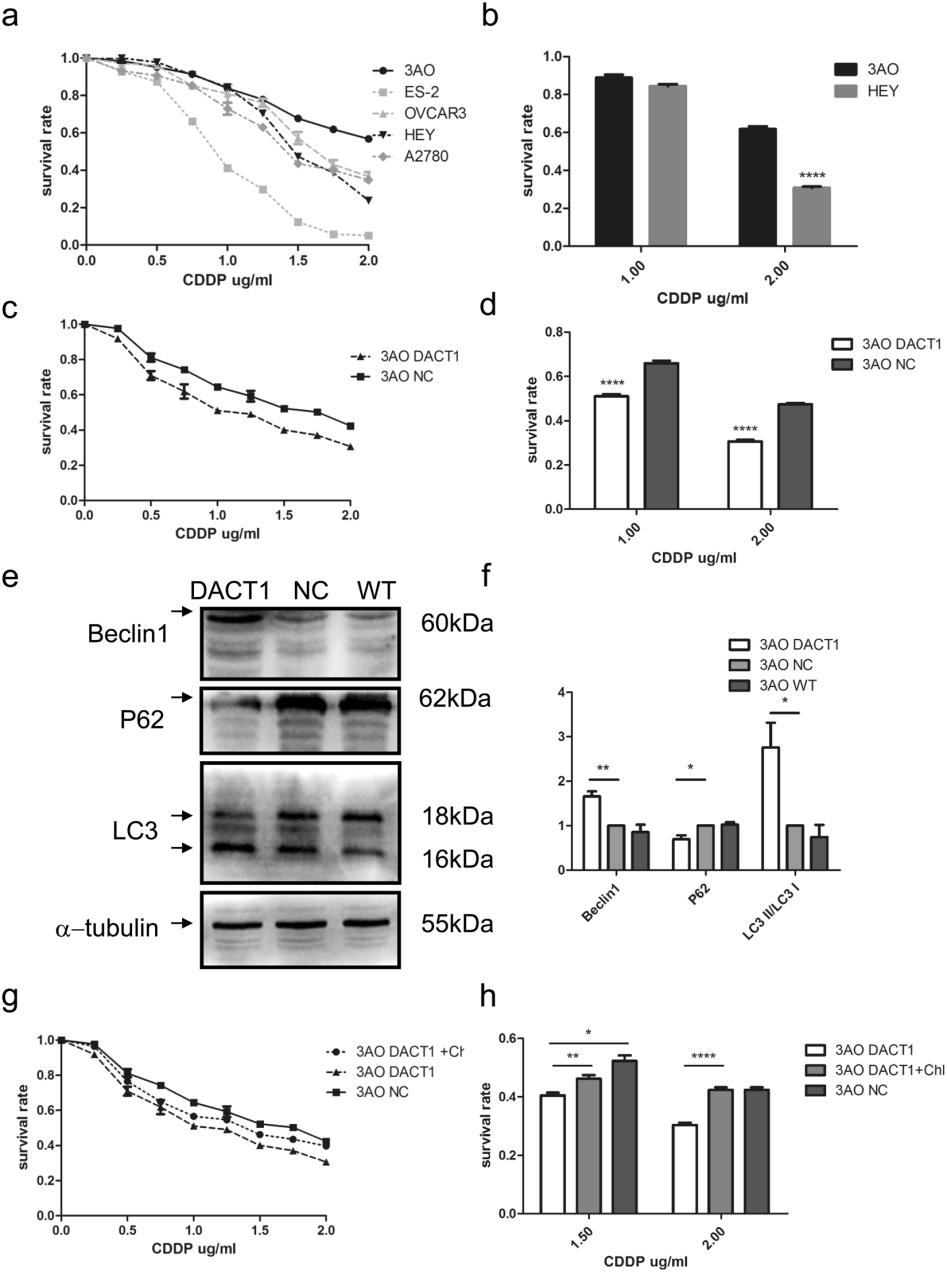
DACT1 expression increased 3AO chemoresponse to cis-platinum by enhancing autophagy. (**a**) and (**b**) Viability of ovarian cancer cell lines incubated with cis-platinum (CDDP), measured by CCK-8 assay. (****P < 0.0001). (**c**) and (**d**) Viability of 3AO-DACT1 or 3AO-NC cells incubated with the indicated concentrations of cis-platinum (****P < 0.0001, paired t test). (**e**) and (**f**) Accumulation of markers of autophagy in 3AO-DACT1 or 3AO-NC cells was assessed by Western blot. Increased levels of Beclin1 and the lipidated form of LC3 (LC3-II/LC3-I) were detected in 3AO-DACT1 cells. α-tubulin was used as the loading control. Grey values collected by Fusion were analysed (*P < 0.05, **P < 0.01). (**g**) and (**h**) Viability of 3AO-DACT1 or 3AO-NC cells incubated with the indicated concentrations of cis-platinum in the presence or absence of chloroquine (***P < 0.001, paired t test, P < 0.05, **P < 0.001, ****P < 0.0001). All the experiment was repeated at least three times.

We first assessed the response of various EOC cell lines to cis-platinum, and measured viability using the CCK-8 assay (Fig. 5a,b). As expected, EOC cell lines with different backgrounds response differently to cis-platinum. We found that 3AO, a typical mEOC cell line, was resistant to cis-platinum, while HEY, a cell line detached from a high grade serous ovarian cancer tissue, was relatively sensitive to cis-platinum. We thus compared the survival ratio between 3AO-DACT1 incubated with cis-platinum (Fig. 5c,d) and found that DACT1 expression increased its sensitivity to cis-platinum.

We measured the expression of P62, Beclin1 and lipidated LC3 (LC3-II)/LC3-I protein by Western Blot (Fig. 5e,f). 3AO-DACT1 contained higher levels of LC3-II and Beclin1, and lower levels of p62/SQSTM1. We then tested whether chloroquine, an autophagy inhibitor, could affect the cell survival of 3AO-DACT1 and 3AO-NC cells. However, there was no significant difference in cell survival with autophagy blocked (Supplementary Fig. S7). To further examine the role of autophagy in cis-platinum resistance, 3AO-DACT1 cells were pre-treated with autophagy inhibitor chloroquine (Chl). As expected, chloroquine partially rescued cis-platinum resistance of 3AO-DACT1 (Fig. 5g,h). Thus these observations implied that DACT1 expression might be pivotal in enhancing sensitivity of mEOC to cis-platinum by induction of autophagy.

## Discussion

As a typical example of type I EOC, mucinous ovarian carcinomas, accounts for 10% of EOC and usually appears as unilateral and expansive tumours mainly featured less aggressiveness^29^. More than half of mEOC patients were diagnosed early (FIGO I-II) and could be removed completely at initial surgery. However, there did have some cases with mEOC were found at advanced stages (FIGO III-IV) in clinical practice. Due to initial resistance to platinum based chemotherapy, those with suboptimal cytoreductive surgery carried even poorer survival in contrast with the same stage type II high grade serous cancer patients. And thus new therapeutic approaches are being needed for these patients. DACT1 is a negative regulator of Wnt signalling via promotion of Dvl degradation^16, 17^. In this study we sought to investigate whether DACT1 is involved in type I EOC, especially in mEOC.

We found that expression of DACT1 was lower in EOC cell lines than normal ovarian tissues, and further that DACT1 expression was significantly lower in type I EOC cell lines, which include 3AO, a mEOC cell line arising from a patient with primary mucinous ovarian cancer. Aberrant down regulation of DACT1 was previously observed in hepatocellular carcinoma, gastrointestinal stromal tumours and non-small-cell lung cancer (NSCLC)^18–20^, whereas in colon cancer and squamous cell carcinoma DACT1 was reported to be overexpressed^21, 22^. We attempted to further analyse the association between DACT1 expression and prognosis of mEOC patients by querying the NCI-Cancer Genome Atlas (TCGA) and GEO database for mEOC samples. However, we identified no cases of mEOC preventing analysis, probably on account of its fairly low incidence in clinical practice. Thus the clinical implications of our results should be further confirmed by studies of larger numbers of patients.

Epigenetic disruption of tumour suppressor genes, including promoter methylation and histone modification, is a major mechanism of cancer gene regulation^30^. In HCC^18^, bladder urothelial carcinoma^31^ and gastric cancer^32^, DACT1 was previously reported to be silenced by promoter methylation. The DACT1 promoter was previously reported to contain a typical CpG island^26^, and incubation with Decitabine or together with histone deacetylase inhibitor TSA restored DACT1 expression in EOC cell lines, suggesting that epigenetic modification may directly or indirectly influence the transcription levels of DACT1. However, the methylation level of CpG sites within the promoter of DACT1 were not investigated by MSP and BGS analysis, and future studies will be necessary to determine whether DACT1 promoter CpG island hypermethylation could be used as a biomarker of EOC.

3AO is a typical mEOC cell line which is arised from a patient with primary mucinous adenocarcinoma, and expresses very low endogenous levels of DACT1^25^. 3AO cells were transfected with a lentivirus carrying full-length DACT1 (3AO-DACT1) or a control vector (3AO-NC). Over-expression of DACT1 suppressed cell proliferation of 3AO, and when transplanted into nude mice, 3AO-DACT formed smaller tumours. Flow cytometry analysis revealed that DACT1 could reduce the proliferation of 3AO cells through inhibiting the transition from G1 to S phase of the cell cycle and thus resulting in an accumulation of G0/G1 phase population.

Wnt signalling has been reported to play key roles in the regulation of tumourigenesis^14, 15^. Since aberrant activation of Wnt signalling has been implicated in the pathogenesis of EOC and DACT1 can regulate Wnt signalling, we investigated whether DACT1 could regulate tumour growth by modulating Wnt signalling. We found that 3AO-DACT1 contained significantly lower levels of key mediators of canonical Wnt signalling, such as Dvl2, both total and nuclear β-catenin, GSK-3β with phosphorylated Ser9, and the Wnt/β-catenin target genes, CyclinD1 and C-myc. These data suggested that DACT1 acted in 3AO cells by downregulating Dvl2 expression, and blocking the total β-catenin and β-catenin/TCF target gene, CyclinD1 and C-myc. β-catenin stability is regulated by the APC complex, containing Axin, adenomatous polyposis coli, glycogen synthase kinase 3β and casein kinase 1. Interaction of GSK-3β with Axin in the complex facilitates efficient phosphorylation of β-catenin^33^. Phosphorylated β-catenin is subsequently ubiquitinated, leading to its rapid proteasomal degradation. Our data demonstrated that over-expression of DACT1 enhances the activity of GSK-3β through attenuating the level of phosphorylated GSK-3β at Ser9, which could make lower expression of β-catenin translocated in the nuclear. These results support the finding that DACT1 regulates the subcellular location of β-catenin through enhancing the activity of GSK-3β^34^.

DACT1 was also recently reported to act as a positive regulator, promoting formation of the Beclin1-Vps34-Atg14L complex and enhancing autophagy^23^. Platinum-derived compounds act by disrupting DNA structure, initiating apoptosis where the damage cannot be repaired^35^. Autophagy has been reported to be a putative mechanism of cis-platinum resistance^27, 28^, which can either sensitize cells to cancer killing agents^36^ or confer resistance to cis-platinum^37, 38^. We thus explored whether DACT1 could influence the cis-platinum sensitivity of mEOC by activating autophagy.

We found that 3AO, a kind of mEOC cell line, was more resistant to cis-platinum than HEY, a typical type II EOC cell line, which is consistent with the clinical facts that mEOC is more resistant to cis-platinum^7, 8, 29^. During induction of autophagy, the nonlipidated form of LC3 (LC3-I, 18 kDa) is conjugated with phosphatidylethanolamine (PE), and converted into the lipidated form LC3 (LC3-II, 16 kDa), which is associated with autophagosome biogenesis, and activity of autophagy is associated with changes of the cellular level of LC3-II^39–41^. Additionally the autophagy receptors SQSTM1/P62^42^ and Beclin1^43^ have also been used as markers of autophagy induction. 3AO-DACT1 contained higher levels of LC3-II, Beclin1 and lower levels of p62/SQSTM1 and autophagy inhibitor chloroquine partially rescued cis-platinum resistance of 3AO-DACT1. However, the involvement of other mechanisms should also be considered as cis-platinum sensitivity could be only partially reversed by chloroquine treatment. Overall, these results highlight the role of autophagy in the treatment of type I EOC, especially the management of ovarian mucinous carcinoma.

In this study, we found in mEOC, DACT1 can inhibit Wnt signalling and active autophagy. A mutually regulative relationship between autophagy and Wnt signalling has been reported^44, 45^ and it needs further study to testify whether it be involved in the regulatory role of DACT1 in tumour growth and cis-platinum resistance.

In conclusion, our results suggest that DACT1 expression is depressed in EOC probably by methylation, for the depressed expression could be reversed by pharmacological demethylation. Differences in the level of DACT1 expression between type I and II EOC suggest that this protein plays a key role in the specific behaviour of type I EOC. Overexpression of DACT1 suppressed proliferation of a mEOC cell line, likely by enhancing GSK3β and inhibiting Wnt/β-catenin signalling. In addition, overexpression of DACT1 increased its chemosensivity to cis-platinum, likely by enhancing autophagy activity. Our findings suggest that DACT1 functions as a tumour suppressor in type I EOC and highlight a potential new biomarker of cis-platinum response and a novel therapeutic target against type I EOC, especially against mEOC.

## Materials and Methods

### Tissue specimens

Primary type I EOC tissue samples were obtained from 49 patients that underwent surgery between August, 2013 and August, 2015 at the Department of Obstetrics and Gynaecology of the First Affiliated Hospital of Chongqing Medical University, China. The patients with other kind of malignancies were excluded. And those who had undergone chemotherapy or radiation previously were not included. The normal ovarian tissues were obtained from 10 patients with benign disease (adenomyosis, adenoma) who chose hysterectomy and oophorectomy. Diagnosis was confirmed by histopathology in all cases. Three patients had low grade serous ovarian cancer, 22 clear cell carcinoma, 6 low grade endometrioid adenocarcinoma and 18 mucinous carcinomas. The clinical information of the cases were presented in Supplementary Table 1.

### Cell culture

SKOV3 was purchased from the American Type Culture Collection (Manassas, VA, USA), and ES-2, OVCAR3, 3AO, A2780 were acquired from the Chinese academy of sciences cell bank (Shanghai, China), while Hey were kindly provided by GeneChem (Shanghai, China). All cell lines were maintained in RMPI 1640 culture medium (Sigma, USA) supplemented with 10% foetal bovine serum (Kangwei, China) and 1% penicillin-streptomycin antibiotics (Beyotime, China) in a humidified (5% CO_2_, 95% air, 37 °C) incubator.

### Decitabine and trichostatin A treatment

Cell lines were treated with 10 mM Decitabine (Aza, Sigma-Aldrich, St Louis, MO, USA) for 72 h in the absence or presence of 100 nM trichostatin A (TSA; Cayman Chemical Co., Ann Arbor, MI, USA) for 24 h as previously described^46^.

### DACT1 expression in ovarian cancer cell lines

A lentivirus carrying full-length DACT1 DNA sequence was constructed by GeneChem (Shanghai, China). 1 * 10^5^ cells were seeded per well in 6-well plates and next day the medium was replaced with 4 * 10^6^ lentivirus (MOI 40) in 1 mL serum-free 1640 with 1 μg/ml polybrane. 24 hours later, the medium was replaced with complete medium. Stable DACT1 expressing cells were selected by incubation with 1 mg/ml puromycin for 72 h.

### Quantitative reverse transcription polymerase chain reaction

Total RNA was isolated from cell lines and tissues using TRIZOL (TAKARA, Japan) according to the manufacturer’s protocol, and DACT1 expression was assessed by quantitative Real-time RT-PCR using the following primers: sense, 5′-GACGAGCAGAGCAATTACACC-3′; antisense, 5′-ACCGTTTGAATGGGCAGA-3′. DACT1 expression was normalized to β-actin expression using the comparative CT-method^47^ with the following primers: sense, 5′-CCTGTGGCATCCACGAAACT-3′; antisense, 5′-GAAGCATTTGCGGTGGACGAT-3′. All reactions were performed in triplicate with the Maxima SYBR Green qPCR Master Mix (TAKARA, Japan) in 10 μl with 5 μl SYBR, 0.4 μl of each primer (10 μM), 3.2 μl DEPC treated water and 1000 ng cDNA, using the Bio-RAD CFX96 Real-Time System at 95 °C for 30 s, 40 cycles of 95 °C for 5 s, 58 °C for 30 s and 72 °C for 15 s.

### Colony-formation assay

Colony-formation assay was performed using a monolayer culture. Cells were re-suspended in RMPI 1640 and 500 cells were seeded per well in 24-well plates. Plates were incubated at 37 °C for 12 days, and colonies were stained with crystal violet (0.005%; Sigma, USA). All experiments were performed in triplicate.

### MTT assay

3AO-DACT1 and 3AO-NC cells were seeded in 96-well plates at the density of 200 cells/well in 200 μl RMPI and 20 μl MTT reagent (5%). At 0,1,2,3,4,5,6 and 7 day, cell proliferation was measured by MTT assay. The absorbance (OD value) was measured at the wavelength of 570 nm by the Microplate Reader (Thermo Fisher, USA). All experiments were performed in triplicate.

### CCK-8 assay

Cells were seeded in 96-well plates at the density of 5000 cells/well in 200 μl RMPI 1640 with 10 μl cis-platinum with stepwise concentrations (from 0 to 2 μg/ml). 72 h later the medium was replaced with 100 μl RMPI 1640 and 10 μl CCK-8 reagent original fluid (Dojindo, Japan). The absorbance (OD value) was measured at 450 nm by the Microplate Reader (Thermo Fisher, USA). All experiments were performed in triplicate.

### Western blot

3AO-DACT1 and 3AO-NC cells were harvested and lysed in RIPA Lysis Buffer (Beyotin, China) with PMSF (1%). To assess the subcellular localization of proteins, nuclear protein was extracted by the Nuclear Protein Extraction Kit (Bestbio, China) according to the instructions. Protein samples were incubated for 30 min on ice and cell debris was removed by centrifugation. The supernatant, containing 15 µg of total protein lysate from each sample, was subjected to sodium dodecyl sulphate-polyacrylamide gel electrophoresis (SDS-PAGE) and transferred onto PVDF membranes, which were probed with anti-DACT1 (1:1000), anti-β-catenin (1:1000), anti-Dvl2 (1:750), (Abcam, Cambridge, UK); anti-CyclinD1 (1:1000), anti-C-myc (1:1000), anti-GSK3β, anti-p-GSK3β (Cell Signalling Technology, USA); anti-P62 (1:1500), anti-LC3 (1:1000), (Sigma, USA); anti-Beclin1 (1:1000), (Abcam, Cambridge, UK), anti-α-tubulin (1:2000) (Beyotime, China) primary antibody at 4 °C overnight. After rinsing in TBST three times for 30 min, the membrane was incubated with 1:2000 goat anti-rabbit or mouse horseradish-peroxidase coupled secondary antibody (Dingguo Changsheng, China) for 2 h at room temperature. Protein bands were visualized by enhanced chemilluminescence detection system (Beyotime, China) with the Chemiluminescence imaging system (Vilber Fusion FX5, France) and grey values were measured by Fusion. All experiments were performed in triplicate.

### Immunohistochemistry

Formalin-fixed, paraffin-embedded tissue sections were dewaxed with xylene and dehydrated by graded alcohol, then permeabilized by incubation in TritonX-100 (0.3%) for 10 min. Endogenous peroxidase activity was blocked with incubation in 3% H_2_O_2_ for 15 min at 37 °C. After antigen retrieval in citrate buffer, all sections were blocked with normal goat serum for 30 min to eliminate nonspecific binding, samples were incubated with DACT1-directed antibody (dilution 1:200, Abcam, Cambridge, UK) overnight at 4 °C, then biotinylated anti-rabbit secondary antibody for 30 min at 37 °C. Samples were rinsed in PBS then incubated with SABC reagent (Boster, China) according to the manufacturer’s instructions. Sections were stained by DAB and then counterstained with haematoxylin.

Images were digitally captured at a ×400 magnification ratio using an Olympus BX51 optic microscope (Olympus, Japan). Staining intensity was assessed using a histochemistry score (H-score), as previously described^48^.

### *In vivo* tumour growth

Six-week-old female nude mice (approximately 18 g) were obtained from the Experimental Animal Centre of Chongqing Medical University. All the mice were bred under SPF (Specific pathogen Free) conditions. All the mice were allowed to acclimate for 1 week, and then 5 * 10^6^ 3AO-DACT1 or 3AO-NC cells were injected into the subcutaneous tissue of each nude mouse (n = 5 animals per group) in the subcutaneous group. Tumour size was measured weekly with a Venire calliper, and tumour volumes were calculated according to the following formula: π/6* length * width^2, 49^. In the intraperitoneal injection group, 1 * 10^7^ 3AO-DACT1 or 3AO-NC cells were injected into the peritoneal cavity (n = 6 animals per group), and all mice were sacrificed after 5 weeks. All studies were approved by the Animal Care Committee of Chongqing Medical University.

### Statistical analysis

SPSS 17.0 (SPSS Inc., Chicago, USA) for Windows was used for statistical analysis. Quantitative data is expressed as mean ± SD and analysed by T-test. Groups were compared using One-way ANOVA with post-hoc method using the S-N-K method. P values < 0.05 were considered to indicate statistical significance. Abnormal distribution data was expressed as median (minimum- maximum) and analysed by Mann-Whitney U test.

### Ethical statement

We solemnly stated that all experiments were repeated at least three times and all methods were performed in accordance with the relevant guidelines and regulations. All clinical investigations have been conducted according to the tenets of the Declaration of Helsinki. All human and animal studies have been approved by the institutional Clinical Research Ethics Review Committee of Chongqing Medical University. Written informed consent was obtained from each patient prior to inclusion in the study.

## Electronic supplementary material

Supplementary Figures

Supplementary Table 1
