# Author Correction: DACT1 Overexpression in type I ovarian cancer inhibits malignant expansion and cis-platinum resistance by modulating canonical Wnt signalling and autophagy

**DOI:** 10.1038/s41598-025-11377-0

**Published:** 2025-08-19

**Authors:** Ruo-nan Li, Bin Liu, Xue-mei Li, Liang-si Hou, Xiao-ling Mu, Hui Wang, Hua Linghu

**Affiliations:** 1 Department of Obstetrics and Gynaecology, the First Affiliated Hospital of Chongqing Medical University, Chongqing, 400016 China; 2 Experimental Research Centre, the First Affiliated Hospital of Chongqing Medical University, Chongqing, 400016 China; 3 Molecular Oncology and Epigenetics Laboratory, the First Affiliated Hospital of Chongqing Medical University, Chongqing, 400016 China; 4 Department of Pathology, the Basic Medical School of Chongqing Medical University, Chongqing, 400016 China; 5 Department of Gynaecologic Oncology, Anhui Provincial Cancer Hospital, Hefei, 230031 China

Correction to: *Scientific Reports* 10.1038/s41598-017-08249-7, published online 24 August 2017

This Article contains errors.

As a result of an error during figure assembly an incorrect image for internal reference strip tubulin blot was used in Figure 2A.

The correct Figure 2 and accompanying legend appear below.

**Fig. 2:**
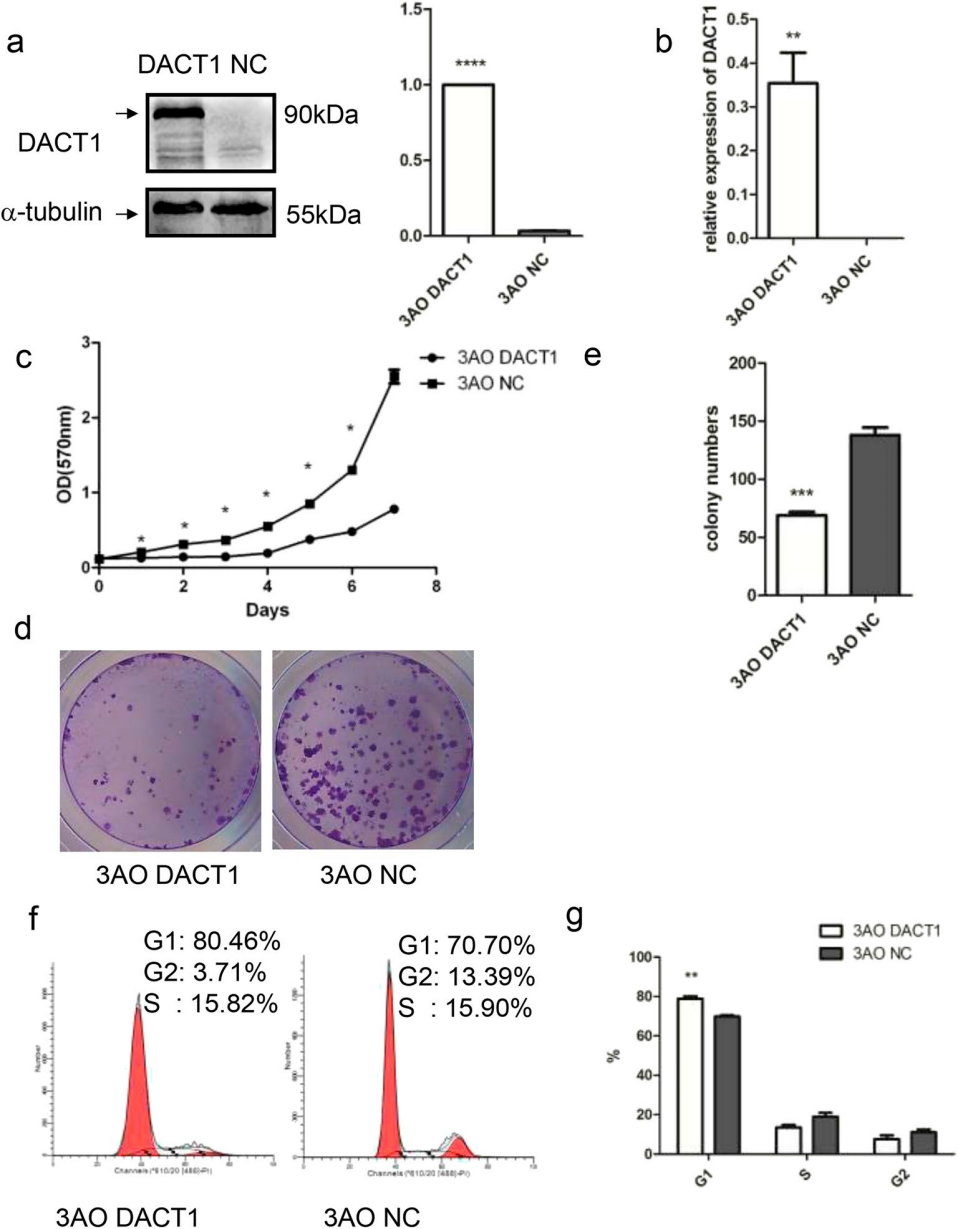
Overexpression of DACT1 inhibition of cell growth and clonogenicity in 3AO cell line. (**a**) Representative Western blots of negative control-transfected (NC) and DACT1-transfected 3AO cells (DACT1), a typical type I EOC cell line with clear genetic background which arises from a patient with primary mucinous adenocarcinoma. α-tubulin was used as the loading control (****P < 0.0001). (**b**) The level of DACT1 mRNA expression in 3AO-DACT1 cells and 3AO-NC cells was detected by quantitative real-time RT-PCR (**P < 0.01). (**c**) Growth curve of 3AO-DACT1 cells and 3AO-NC cells (p < 0.05, paired t test). Cell numbers were assessed by MTT assay from days 0 to 7. (**d**) and (**e**) The effect of DACT1 overexpression on 3AO cell colony formation. 3AO-DACT1 or 3AO-NC were incubated for 12 days and colonies of over 50 cells were counted after staining with crystal violet. (***P < 0.001). (**f**) The effect of DACT1 on 3AO cell cycle was assessed by flow cytometry (**P < 0.01). All the experiment was repeated at least three times.

