# Supplementary material for: DACT1 Overexpression in type I ovarian cancer inhibits malignant expansion and cis-platinum resistance by modulating canonical Wnt signalling and autophagy
Source: Sci Rep. 2017 Aug 24;7:9285. doi: 10.1038/s41598-017-08249-7 (PMC5570946; doi:10.1038/s41598-017-08249-7)
Supplement: Supplementary file 1 — Supplementary Figures [file 41598_2017_8249_MOESM1_ESM.pdf]

DACT1 Overexpression in Type I ovarian cancer inhibits malignant expansion and cis-platinum resistance by modulating canonical Wnt signalling and autophagy

Ruo-nan Li[1,2,3,5], Bin Liu[4], Xue-mei Li[1,2], Liang-si Hou[1,2], Xiao-ling Mu[1], Hui Wang[1], Hua Linghu[1*]

[1]Department of Obstetrics and Gynaecology, the First Affiliated Hospital of Chongqing Medical University, Chongqing 400016, China

[2]Experimental Research Centre, the First Affiliated Hospital of Chongqing Medical University, Chongqing 400016, China

[3] Molecular Oncology and Epigenetics Laboratory, the First Affiliated Hospital of Chongqing Medical University

[4]Department of Pathology, the Basic Medical School of Chongqing Medical University, Chongqing 400016, China

[5]Department of Gynaecologic Oncology, Anhui Provincial Cancer Hospital, Hefei, 230031, China

***Corresponding author:**

Hua Linghu

Department of Obstetrics and Gynaecology,

The First Affiliated Hospital of Chongqing Medical University,

Chongqing 400016, China

Tel: +86-23-89011090

Fax: +86-23-68811487

E-mail: linghu_hua@yahoo.com

Supplementary figures

Fig.S1 DACT1 mRNA expression in type I EOC cells was detected by semi-quantitative RT-PCR.

Fig.S2 The DACT1 mRNA expression was restored by pharmacologic demethylation, and the results were verified by semi-quantitative RT-PCR. (WT: wild type; A: Aza; A+T: Aza with TSA)

Fig.S3 Two representative sections of type I ovarian cancer tissue which included normal ovarian tissue, borderline lesion and mucinous cystadenocarcinoma were provided.

Fig.S4 Wild type 3AO cells were injected into the subcutaneous tissue, and the xenograft tumour was removed to perform the HE staining to validate the histologic classification of 3AO.

Fig.S5 $1*10^7$ 3AO-DACT1 or 3AO-NC cells were injected into the peritoneal cavity of the nude mice, and nude mice were mercy killed after 5 weeks to analyze the tumourigenesis of 3AO-DACT1 and 3AO-NC. (a) Photos of the two groups of nude mice. (b) General conditions and the metastasis statistics between the 3AO-DACT1 (n=6) and 3AO-NC groups (n=6).

Fig.S6 Representative immunofluorescence analysis of β-catenin expression in 3AO-DACT1 and 3AO-NC cells. (Scale bar: 25μm). Blue: DAPI, Red: β-catenin.

Fig.S7 3AO-DACT1 and 3AO-NC cells were incubated with the autophagy inhibitor chloroquine for 24h, and then cell survival ratio of each group was measured by CCK-8 assay (ns, $p>0.05$).The experiment was repeated at least three times.

Supplementary figures

Fig.S1

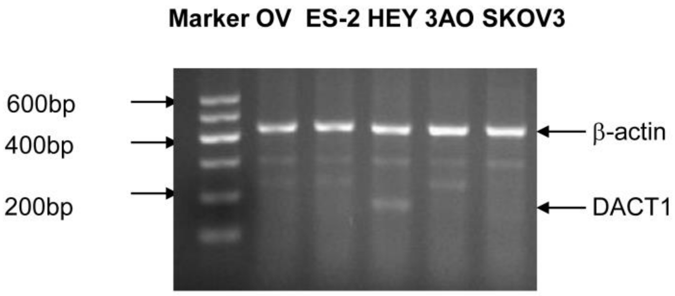

Fig.S2

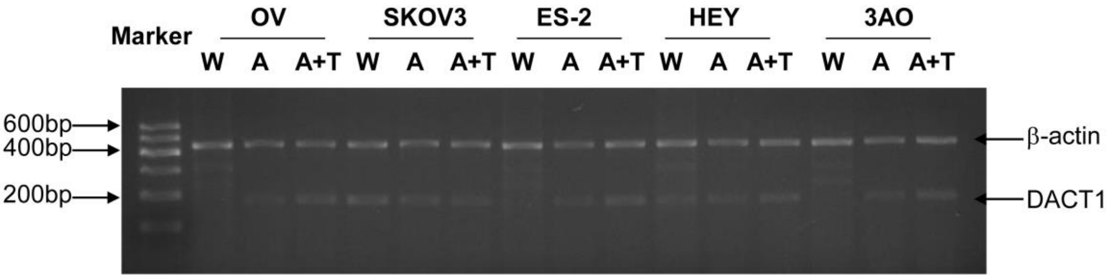

Fig.S3

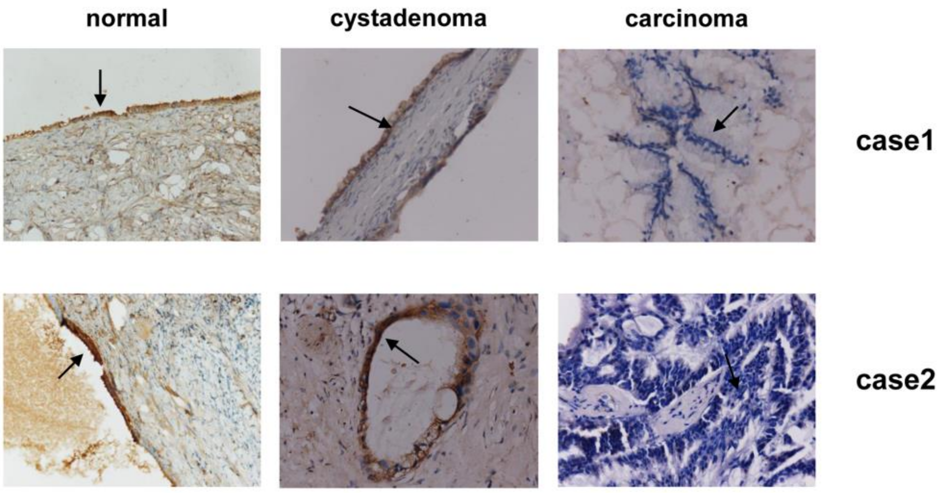

×400

Fig.S4

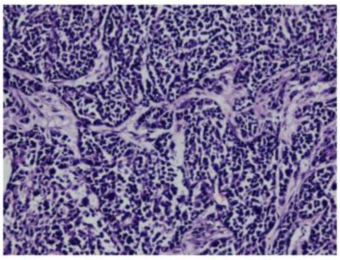

3AO Wild Type×400

Fig.S5

a

3AO NC

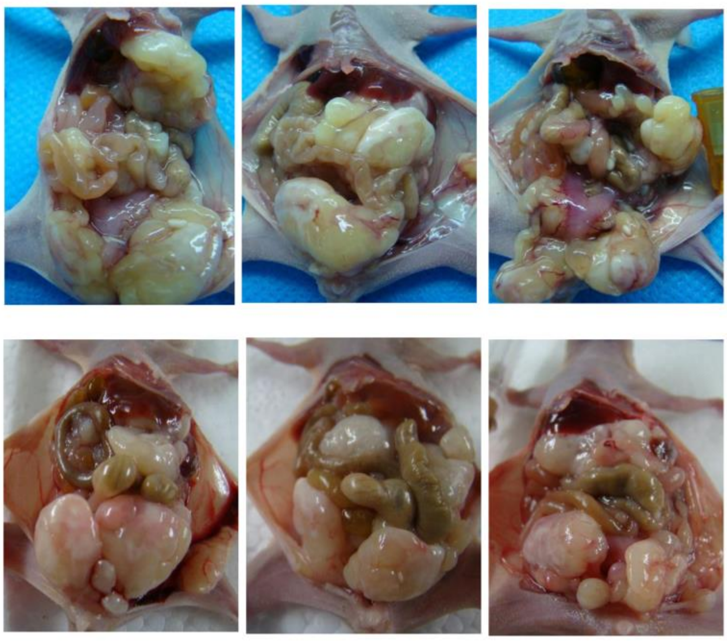

3AO DACT1

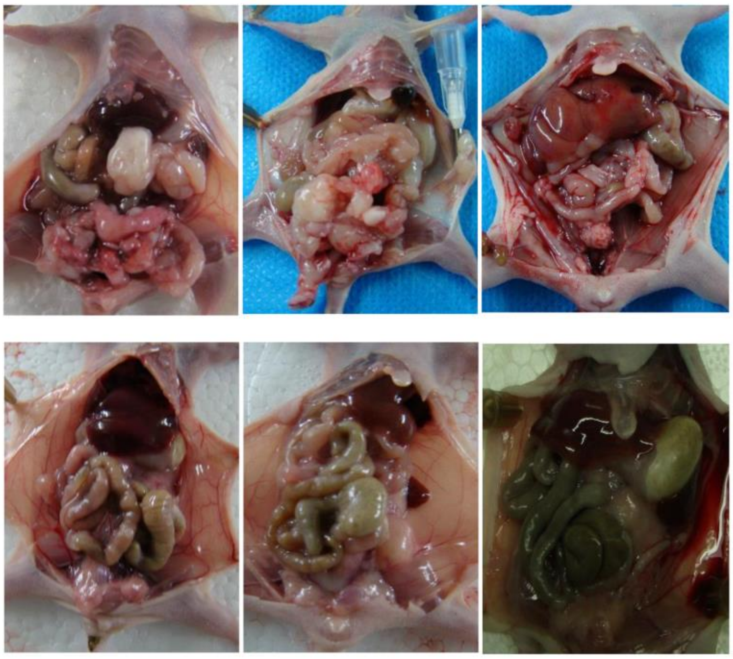

b

	3AO NC	3AO DACT1
tumourigenesis	6/6	2/6
cachexia	6/6	1/6
ascites	4/6	0/6

	3AO NC	3AO DACT1	P value
Cancer lesions on ovary and uterus	36.33±5.50	1.83±2.86	<0.0001
Hepatic metastasis	6.67±3.93	2.33±3.61	0.0744
Gastrointestinal metastasis	49.00±10.51	7.67±12.29	<0.0001
Splenic metastasis	5.67±4.80	1.00±1.55	0.0467
Kidney metastatic	7.00±2.52	2.83±4.40	0.0716
Diaphragm metastasis	13.67±2.50	3.00±4.69	0.0006

Fig.S6

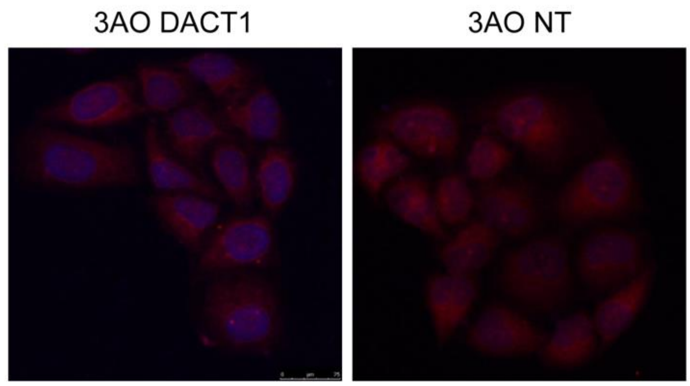

3AO DACT1
3AO NT

Fig.S7

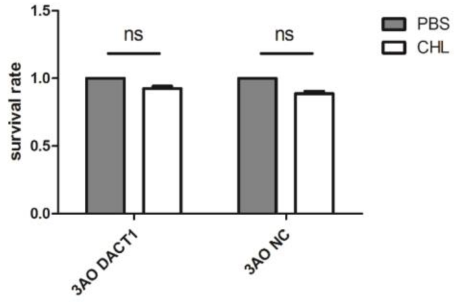

survival rate
1.5
1.0
0.5
0.0
ns
ns
PBS
CHL
3AO DACT1
3AO NC
