# Supplementary material for: DACT1 Overexpression in type I ovarian cancer inhibits malignant expansion and cis-platinum resistance by modulating canonical Wnt signalling and autophagy
Source: Sci Rep. 2017 Aug 24;7:9285. doi: 10.1038/s41598-017-08249-7 (PMC5570946; doi:10.1038/s41598-017-08249-7)
Supplement: Supplementary file 2 — Supplementary Table 1 [file 41598_2017_8249_MOESM2_ESM.pdf]

DACT1 Overexpression in Type I ovarian cancer inhibits malignant expansion and cis-platinum resistance by modulating canonical Wnt signalling and autophagy

Ruo-nan Li[1,2,3,5], Bin Liu[4], Xue-mei Li[1,2], Liang-si Hou[1,2], Xiao-ling Mu[1], Hui Wang[1], Hua Linghu[1*]

[1]Department of Obstetrics and Gynaecology, the First Affiliated Hospital of Chongqing Medical University, Chongqing 400016, China

[2]Experimental Research Centre, the First Affiliated Hospital of Chongqing Medical University, Chongqing 400016, China

[3] Molecular Oncology and Epigenetics Laboratory, the First Affiliated Hospital of Chongqing Medical University

[4]Department of Pathology, the Basic Medical School of Chongqing Medical University, Chongqing 400016, China

[5]Department of Gynaecologic Oncology, Anhui Provincial Cancer Hospital, Hefei, 230031, China

***Corresponding author:**

Hua Linghu

Department of Obstetrics and Gynaecology,

The First Affiliated Hospital of Chongqing Medical University,

Chongqing 400016, China

Tel: +86-23-89011090

Fax: +86-23-68811487

E-mail: linghu_hua@yahoo.com

Supplementary Table1

The clinical and histological characteristics and the H-score of the type I EOC cases.

Case number	Age (year)	Histological type	FIGO stage	H-score
1	46	CCC	II	8
2	48	MC	II	8
3	38	CCC	II	1
4	56	LGEC	Ia	4
5	56	CCC	Ia	4
6	60	CCC	IIIc	0
7	54	CCC	II	4
8	49	CCC	II	2
9	40	CCC	II	4
10	61	CCC	IIIb	4
11	44	CCC	Ia	3
12	40	CCC	II	4
13	45	CCC	Ic	6
14	44	CCC	II	4
15	42	CCC	I	6
16	42	CCC	I	2
17	17	MC	Ia	3
18	55	CCC	Ic	4
19	44	LGEC	I	4
20	47	MC	IIIc	8
21	44	LGEC	I	1
22	44	LGEC	II	3
23	58	MC	IIIc	4
24	48	CCC	IV	6
25	61	MC	IIIc	8
26	34	LGSC	Ib	4
27	52	CCC	IIIc	1
28	45	CCC	Ia	4
29	44	LGEC	I	8
30	51	CCC	IV	8
31	57	CCC	II	2
32	52	LGSC	IIIc	8
33	14	MC	Ia	2
34	45	CCC	Ia	3
35	41	LGSC	IIc	1
36	51	CCC	IIc	8

37	46	LGEC	IIc	4
38	46	MC	IIb	4
39	25	MC	I	0
40	55	MC	IIa	0
41	79	MC	III	0
42	39	MC	IIb	0
43	40	MC	IV	0
44	47	MC	II	0
45	73	MC	IV	0
46	36	MC	IV	0
47	60	MC	III	0
48	54	MC	IIIc	0
49	26	MC	Ia	0

Abbreviation:
LGEC: low grade endometrial carcinoma
CCC: clear cell carcinoma
MC: mucinous carcinoma
LGSC: low grade serous carcinoma
